# Pacifier use and breastfeeding in term and preterm newborns—a systematic review and meta-analysis

**DOI:** 10.1007/s00431-022-04559-9

**Published:** 2022-07-14

**Authors:** Olli Tolppola, Marjo Renko, Ulla Sankilampi, Panu Kiviranta, Leena Hintikka, Ilari Kuitunen

**Affiliations:** 1 Institute of Clinical Medicine and Department of Pediatrics, University of Eastern Finland, Kuopio, Finland; 2 Department of Pediatrics, Kuopio University Hospital, Kuopio, Finland; 3 Department of Pediatrics, Mikkeli Central Hospital, Perhetalo 3rd floor, Porrassalmenkatu 35–37, 50100 Mikkeli, Finland

**Keywords:** Breastfeeding, Pacifier, Intervention, Meta-analysis, Non-nutritive sucking

## Abstract

The purpose of this study is to assess whether pacifier use is associated with breastfeeding success in term and preterm newborns and whether it influences hospitalization time in preterm newborns. Four databases were searched for randomized controlled trials (RCTs), and a systematic review and meta-analysis were conducted. The risk of bias and evidence quality, according to the GRADE methodology, were analyzed. Risk ratios with 95% confidence intervals (CI) for dichotomous outcomes and mean difference (MD) for continuous outcomes were used. The random effect model was used if heterogeneity was high (*I*^2^ over 40%). We screened 772 abstracts, assessed 44 full texts, and included 10 studies, of which 5 focused on term and 5 on preterm newborns. There were a few concerns about the risk of bias in 9 of the 10 studies. Breastfeeding rates were analyzed at 2, 3, 4, and 6 months, and the success rates were similar between the restricted and free pacifier use groups (evidence quality was moderate to high). In preterm neonates, the use of a pacifier shortened the duration of hospitalization by 7 days (MD 7.23, CI 3.98–10.48) and the time from gavage to total oral feeding by more than 3 days (MD 3.21 days, CI 1.19–5.24) (evidence quality was ranked as moderate).

*   Conclusions*: Based on our meta-analysis, pacifier use should not be restricted in term newborns, as it is not associated with lower breastfeeding success rates. Furthermore, introducing pacifiers to preterm newborns should be considered, as it seems to shorten the time to discharge as well as the transition time from gavage to total oral feeding.

**Table:** 

**What is Known:** *• Observational studies show that infants who use a pacifier are weaned from breastfeeding earlier.* *• Previous randomized studies have not presented such results, and there have been no differences in the successful breastfeeding rates regardless of the use of pacifier.*	
**What is New:** *• Term and preterm newborns do not have worse breastfeeding outcomes if a pacifier is introduced to them, and additionally preterm newborns have shorter hospitalization times.* *• The decision to offer a pacifier should depend on the caregivers instead of hospital policy or staff recommendation, as there is no evidence to support the prohibition or restriction.*

**Supplementary Information:**

The online version contains supplementary material available at 10.1007/s00431-022-04559-9.

## Introduction

Breastfeeding has many benefits for both infants and their mothers and should therefore be encouraged. Previous meta-analyses have shown that breastfeeding has short- and long-term benefits for children. The short-term benefits of breastfeeding include decreased mortality and morbidity since it reduces diarrhea and digestive and respiratory tract infection rates. Breastfeeding also protects children from being overweight and having obesity and type 2 diabetes. Breastfed infants may have higher intelligence quotients later in childhood [1]. For nursing mothers, benefits include protection against breast and ovarian cancer, type 2 diabetes, weight retention, and depression [1]. Infants must learn the sucking technique early for breastfeeding to be successful [2].

In their Baby-Friendly Hospital Initiative “Ten Steps for Successful Breastfeeding,” the World Health Organization (WHO) recommends counseling mothers on the risks of using artificial teats or pacifiers [3, 4]. According to the WHO, mothers should be aware that pacifiers may interfere with their ability to recognize infant feeding cues. It has been suggested that if pacifiers replace sucking, the time an infant stimulates mothers’ breast and, thus, milk production may decrease.

Observational studies have associated early pacifier use with breastfeeding problems leading to early weaning [5–7]. However, randomized controlled trials (RCTs) have not shown a similar negative association between early pacifier use and successful breastfeeding, which suggests that pacifier use may be a sign of breastfeeding problems and not its cause [8–11]. Pacifier use reduces the risk of sudden infant death syndrome, and non-nutritive sucking has shown to increase physiologic stability and nutrition in preterm infants. Thus, the risks and benefits of pacifier use should be carefully assessed [12, 13].

As more RCTs have been conducted since the last Cochrane analyses of pacifier use or non-nutritive sucking and the success of breastfeeding, we decided to update the summary of the evidence [14, 15]. We performed a comprehensive systematic literature review and meta-analysis of randomized trials, comparing the effects of restricted and free pacifier use in the success of breastfeeding preterm and term infants. As a secondary outcome, we analyzed the effect of pacifier use on hospitalization time in preterm infants.

## Methods

### Search strategy

For this systematic review, we used PubMed (MEDLINE), the Cochrane Central Register of Controlled Trials (CENTRAL), Web of Science, and Scopus. The literature search was conducted on October 30, 2021, with the terms: (“pacifier” OR “dummy” OR “soother”) AND (“breastfeed*” OR “lactation”). We used neither language nor time restrictions. The results were then uploaded to Covidence software (Covidence, Melbourne, Australia).

### Inclusion and exclusion criteria

All RCTs and cluster or quasi-randomized trials, regardless of blinding, were included. The trials had to focus on the effects of free or restricted pacifier use in newborns. We had no exclusion criteria regarding prematurity or birthweight in our review. We excluded all observational studies.

### Review process

Two authors (IK and OT) individually screened the abstracts, and conflicts were resolved by a third author (MR) or by consensus. Full texts were then assessed by two authors (IK and OT), and the data were extracted to an Excel spreadsheet. We assessed the risk of bias according to the Cochrane Risk of Bias 2.0 tool and generated the risk of bias plots with the robvis package in R version 4.0.3. We assessed the quality of the evidence using the Grading of Recommendations Assessment, Development and Evaluation (GRADE) methodology [16].

### Outcome measures

Our main outcomes were the rates of any breastfeeding and full breastfeeding during the first 6 months of life, and the outcome was measured at the ages of 2, 3, 4, and 6 months. We stratified the analyses based on gestational age into preterm (less than 37 weeks) and full-term (37 weeks or more) infants. Our secondary outcomes were the duration of hospital stay and the time required to achieve full oral feeding in preterm neonates. In term infants, the intervention in the analyses was restricted pacifier use, and comparisons were made with free pacifier use. In preterm infants, the intervention was to offer pacifiers to the infants, and comparisons were made for restricted use.

### Statistics

Review Manager version 5.4 (The Cochrane Collaboration, London, UK) was used for the meta-analysis. Data analyses were performed according to the Cochrane Handbook for Systematic Reviews guidelines. We calculated risk ratios (RR) with 95% confidence intervals (CI) for dichotomous outcomes. Forest plots are presented for all outcomes. We calculated mean differences (MD) with CIs for continuous outcomes, as all the included studies used the same continuous outcome measurements. We analyzed inconsistency index statistics for heterogeneity, and if *I*^2^ >50%, we used the random effect model; otherwise, we used the fixed effect model.

We have reported our systematic review and meta-analysis according to the Preferred Reporting Items for Systematic Reviews and Meta-Analyses (PRISMA) [17]. The checklist can be found in the supplements.

### Protocol registration

We registered our protocol in Prospero with registration number: CRD42021289589. https://www.crd.york.ac.uk/prospero/display_record.php?ID=CRD42021289589.

## Results

### Study selection

Our initial search retrieved 1481 results, and after the exclusion of duplicates, we screened 772 abstracts. We assessed 44 full texts, and a total of 10 RCTs [8–11, 13, 18–22] met our inclusion criteria and were included in the analysis (Fig. S1).

### Study characteristics

Of the ten studies included, five covered term infants [8–11, 18] and five preterm infants [13, 19–22] (Table S1). In the studies with term infants, the intervention groups were instructed not to offer pacifiers during hospital stay or longer (up to 3 months). In the studies with preterm infants, the intervention groups were given pacifiers during the hospital stay. The background characteristics of the studies and the included newborns are described precisely in the supplementary materials (Tables S1 and S2).

### Risk of bias

Risk of bias was assessed in five domains and overall. One of the included studies had a low risk of bias, and nine studies had some concerns (Fig. S2). The lowest risk of bias was due to the selection of reported results, and most concerns were observed in the bias due to the randomizing process, as the authors described the blinding and concealment process inadequately (Fig. S3).

### Breastfeeding rates among infants at 2, 3, 4, and 6 months of age

**Fig. 1:**
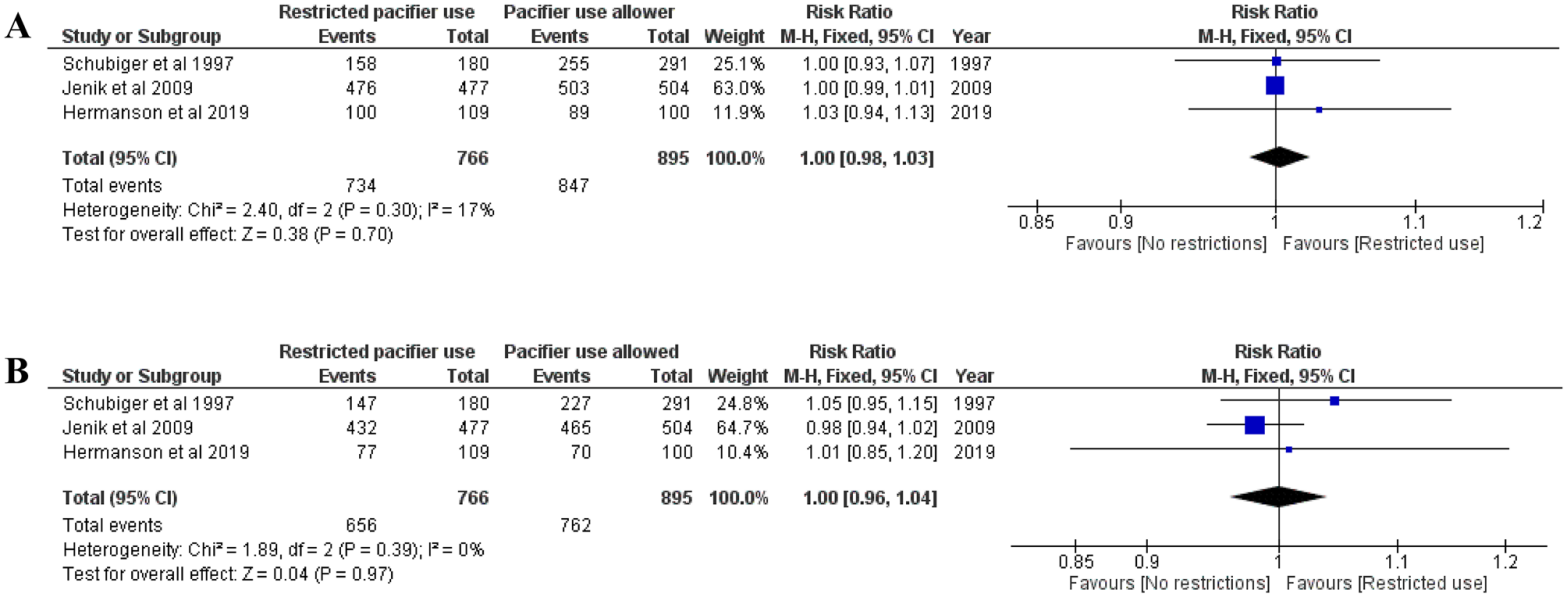
**A** Risk ratio for any breastfeeding at 2 months. Restricted pacifier use compared to no restrictions in pacifier usage. **B** Risk ratio for full breastfeeding at 2 months. Restricted pacifier use compared to no restrictions in pacifier usage

**Fig. 2:**
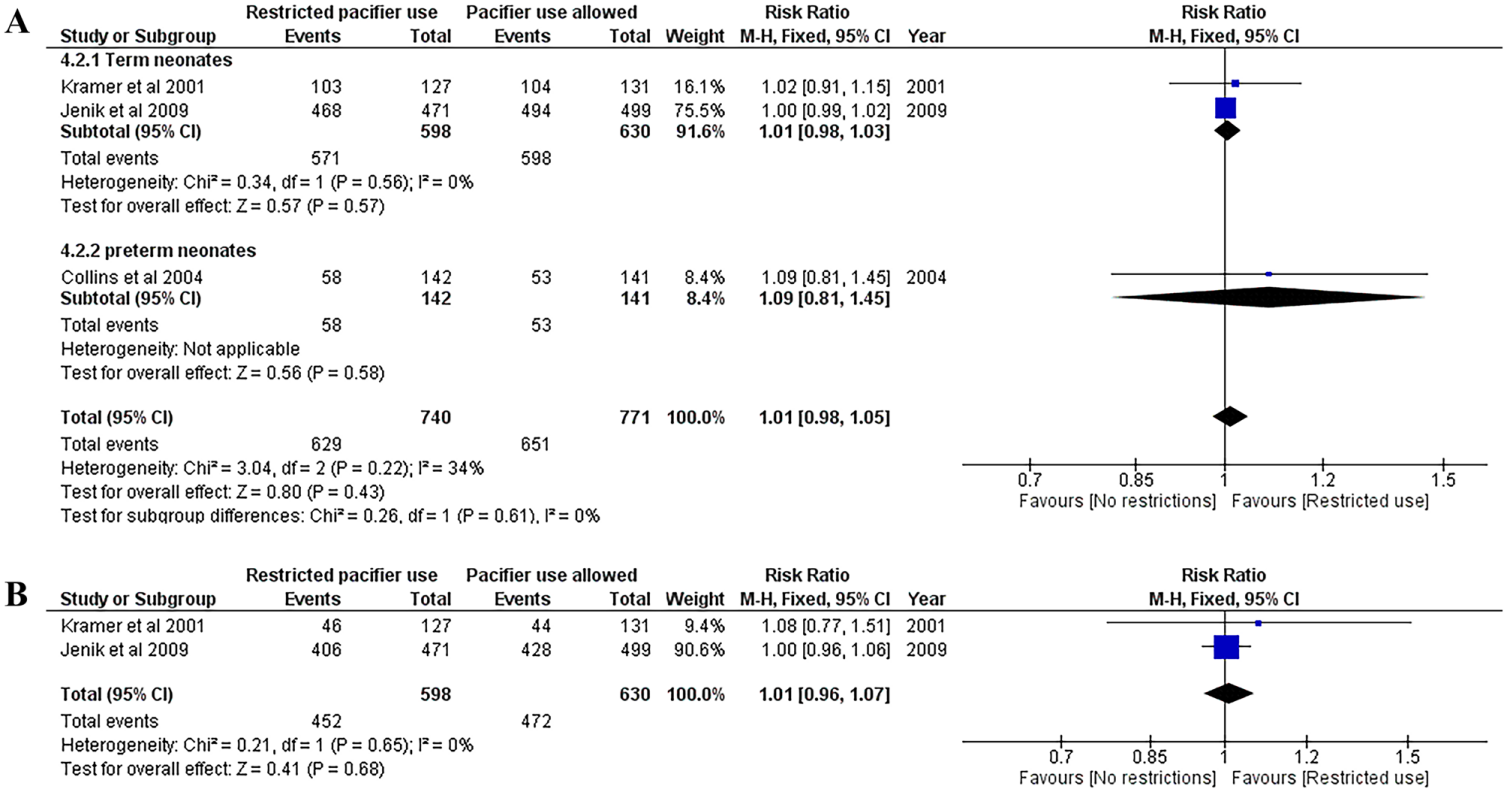
**A** Risk ratio for any breastfeeding at 3 months. Restricted pacifier use compared to no restrictions in pacifier usage. Term and preterm neonates analyzed separately and combined. **B** Risk ratio for full breastfeeding at 3 months. Restricted pacifier use compared to no restrictions in pacifier usage

**Fig. 3:**
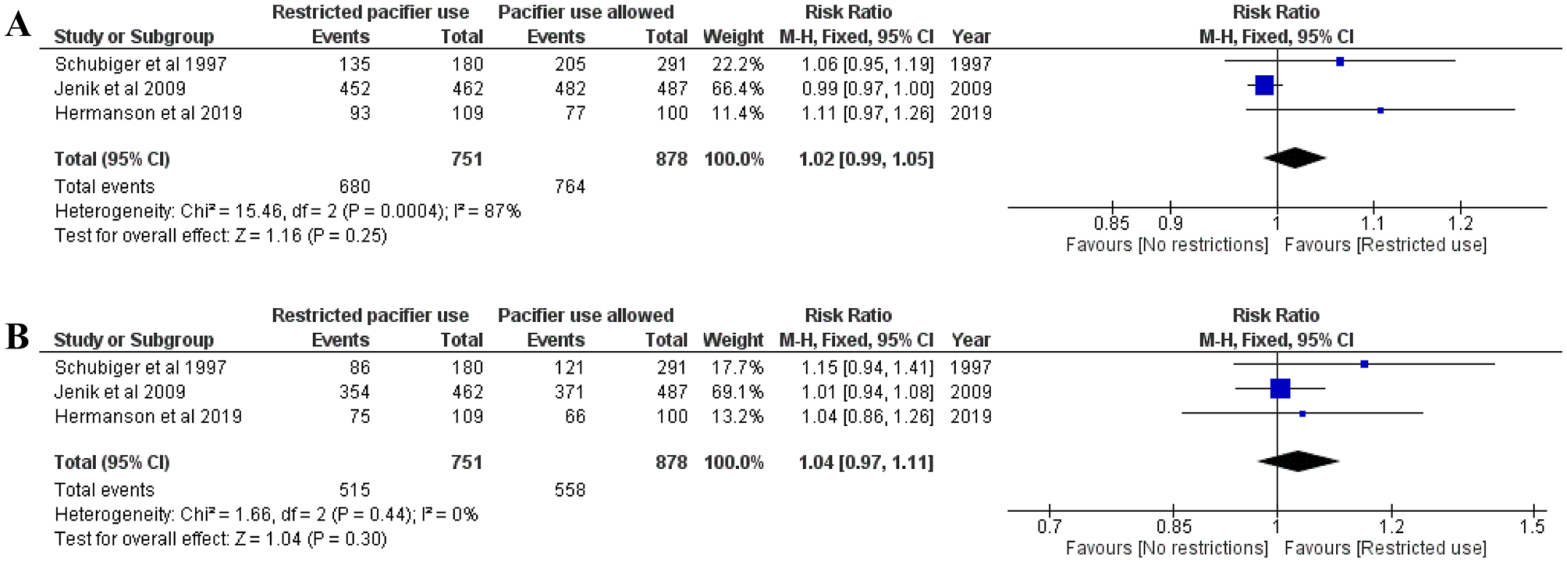
**A** Risk ratio for any breastfeeding at 4 months. Restricted pacifier use compared to no restrictions in pacifier usage. **B** Risk ratio for full breastfeeding at 4 months. Restricted pacifier use compared to no restrictions in pacifier usage

**Fig. 4:**
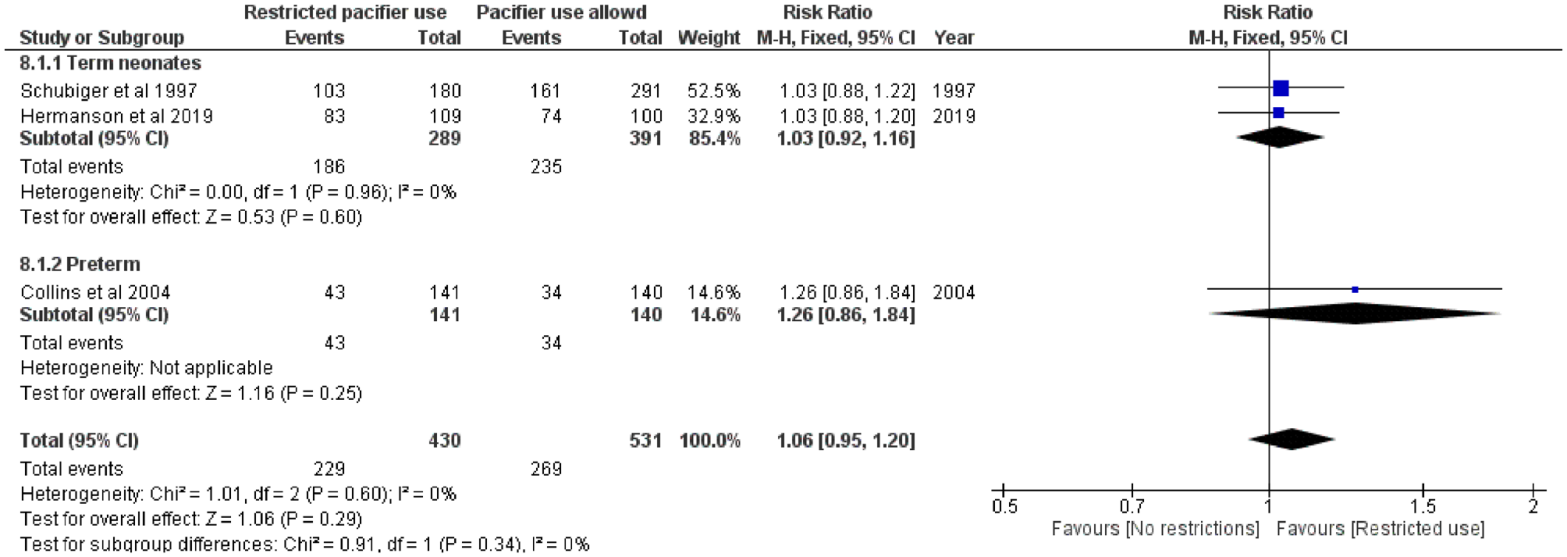
Risk ratio for any breastfeeding at 6 months. Restricted pacifier use compared to no restrictions in pacifier usage. Term and preterm neonates analyzed separately and combined

Three studies [8, 11, 18] that included 1862 term newborns analyzed the rate of any and full breastfeeding at 2 months and reported similar rates between the groups (Fig. 1A–B). Three studies [9, 11, 19] that included 1621 newborns (283 preterm) analyzed the rate of any breastfeeding at 3 months, and two studies [9, 11] that included 1338 term newborns analyzed full breastfeeding at three months and did not report any differences (Fig. 2A–B). Three studies [8, 11, 18] that included 1862 newborns analyzed the rate of full and any breastfeeding at 4 months and reported that the restricted use of pacifiers did not improve breastfeeding rates (Fig. 3A–B). Furthermore, three studies [8, 18, 19] that included 1160 newborns (281 preterm) analyzed the rate of any breastfeeding at 6 months and did not find any significant differences (RR 1.06, CI 0.95–1.20, *I*^2^ = 0 %; Fig. 4). The quality of the evidence was ranked as either moderate or high in these outcomes, and some concerns were noted regarding the risk of bias (Table 1).

**Table 1:** Body of evidence for outcomes assessed by the GRADE methodology

Outcome	Quality assessment								Summary of findings					
									Number of patients			Effect		Evidence quality
	Number of studies	Design	Risk of bias	Inconsistency	Indirectness	Imprecision	Publication bias	Intervention *n*/N		Control *n*/*N*	Relative risk (95% CI)		Absolute risk difference (95% CI)	
Any breastfeeding at 2 months	3	RCT	Some concerns (randomization process and outcome measures)	Low	Not present	Serious limitations: CI includes 1	Not present	734/766		847/895	1.00 (0.98–1.03)		1.0% (−0.9 to 3.0%)	High
Full breastfeeding at 2 months	3	RCT	Some concerns (randomization process and outcome measures)	Low	Not present	Serious limitations: CI includes 1	Not present	656/766		762/895	1.00 (0.96–1.04)		0.5% (−2.9 to 3.9%)	High
Any breastfeeding at 3 months	3	RCT	Some concerns (randomization process and missing outcomes)	Moderate	Not present	Serious limitations: CI includes 1	Not present	629/740		651/771	1.01 (0.98–1.05)		0.5% (−3.1 to 4.2%)	Moderate
Full breastfeeding at 3 months	2	RCT	Some concerns (randomization process and missing outcomes)	Low	Not present	Serious limitations: CI includes 1	Not present	452/598		472/630	1.01 (0.96–1.07)		0.7% (−4.2 to 5.5%)	Moderate
Any breastfeeding at 4 months	3	RCT	Some concerns (randomization process and outcome measures)	Substantial	Not present	Serious limitations: CI includes 1	Not present	680/751		764/878	1.05 (0.91–1.21)		3.5% (−0.5 to 6.6%)	Moderate
Full breastfeeding at 4 months	3	RCT	Some concerns (randomization process and outcome measures)	Low	Not present	Serious limitations: CI includes 1	Not present	515/751		558/878	1.04 (0.97–1.11)		5.0% (0.4 to 9.6%)	High
Any breastfeeding at 6 months	3	RCT	Some concerns (randomization process and outcome measures)	Low	Not present	Serious limitations: CI includes 1	Not present	229/430		269/531	1.06 (0.95–1.20)		2.6% (−3.8 to 9.0%)	High
Hospital stay duration in preterm neonates	4	RCT	Some concerns (randomization process, outcome measures, result selection)	Moderate	Not present	No limitations	Not present	N 127		N 126	N/A		7.23 (3.98–10.48) days, favors pacifier use*	Moderate
Transition time from gavage to oral feeding	4	RCT	Some concerns (randomization process, outcome measures, result selection)	Moderate	Not present	No limitations	Not present	N 127		N 126	N/A		3.21 (1.19–5.24) days, favors pacifier use*	Moderate

^*^Mean difference with 95% CI

### Hospital stay duration among preterm newborns

**Fig. 5:**
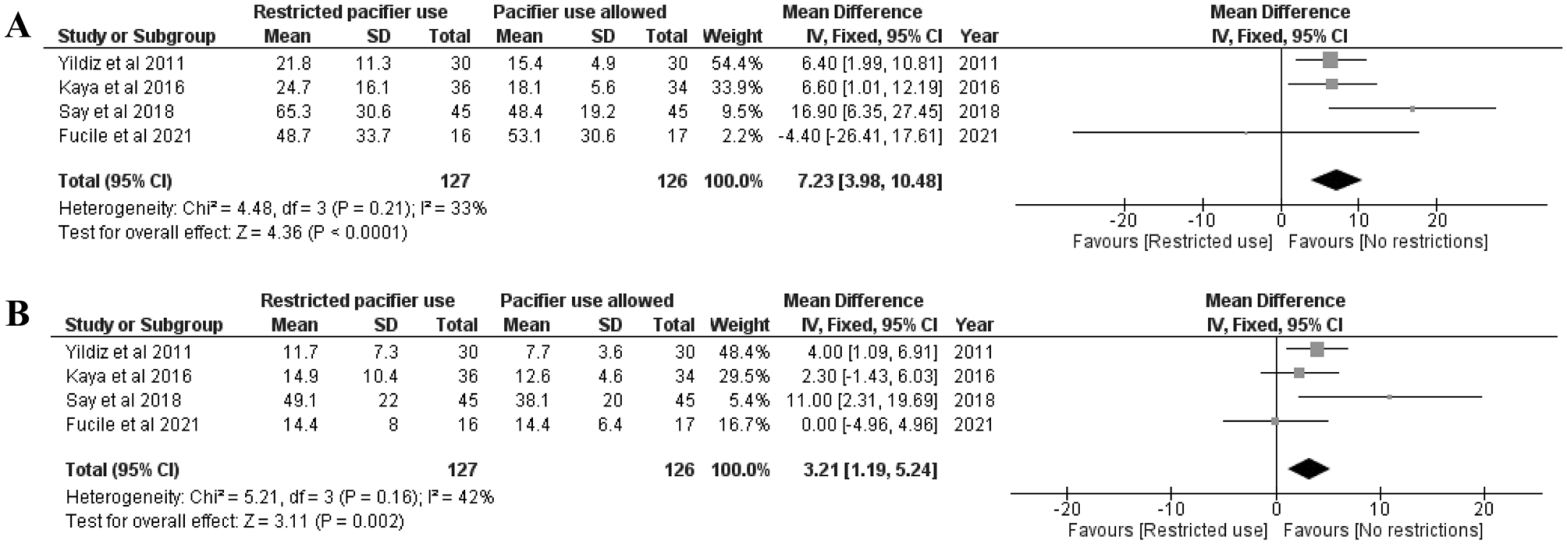
**A** Mean difference in fixed effect model for hospital stay duration in days among preterm neonates. Restricted pacifier use compared to no restrictions in pacifier usage. **B** Mean difference in fixed effect model for time of transition from gavage feeding to total oral feeding. Restricted pacifier use compared to no restrictions in pacifier usage

Four studies [13, 20–22] that included 283 preterm neonates analyzed hospital stay duration. Pacifier use shortened the duration of hospitalization by 7 days (MD 7.23, CI 3.98–10.48, *I*^2^ = 33%, Fig. 5A). We ranked the quality of evidence as “moderate” and risk of bias as “some concern” due to the randomization and outcome measures.

### Transition from gavage feeding to full oral feeding among preterm newborns

Four studies [13, 20–22] that included 283 preterm neonates analyzed the time of transition from gavage feeding to full oral feeding. Pacifier use reduced the time of transition by 3 days (MD 3.21 days, CI 1.19–5.24, *I*^2^ = 42 %, Fig. 5B). The quality of the evidence was ranked moderate, and some concerns were noted as to the risk of bias due to randomization and result selection (Table 1).

## Discussion

In this meta-analysis, we gathered information from 10 RCTs to assess the association between early pacifier use and breastfeeding. We found that early pacifier use was not associated with the duration of partial or exclusive breastfeeding during the first 6 months of life. Furthermore, we found that the length of hospitalization was 7 days shorter and the time from gavage feeding to full oral feeding was 3 days shorter in preterm newborns who used pacifiers in the hospital.

The findings of our meta-analysis are in line with the previous Cochrane analysis in 2016, which indicated that restricted pacifier use does not improve breastfeeding rates [15]. Pacifier use has been associated with lower breastfeeding rates in observational studies but not in any of the randomized studies. This indicates that pacifier use does not have a real causal effect on breastfeeding and that it is rather a sign of breastfeeding problems or a more challenging infant behavior. Therefore, pacifier use should be a caregiver’s decision rather than a policy introduced in maternity hospitals or clinics.

Although observational studies provide insightful and important findings on breastfeeding rates and have shown that breastfeeding rates vary substantially globally, they are generally prone to bias when addressing intervention effectiveness [23]. The role of observational studies is to produce new hypotheses which should, if possible, be tested in RCTs. When it comes to breastfeeding, future research resources should be allocated to study and implement interventions, such as counseling, that could improve breastfeeding rates. [24]

Our findings regarding the shortened time from gavage feeding to full oral feeding and hospitalization time are in line with the previous Cochrane analysis [15]. We included two previous RCTs focusing on pacifiers published after Cochrane analysis in our meta-analysis, and the results did not change. The positive effects of non-nutritive sucking in preterm newborns are clear. The reported reduction in hospitalization time by 7 days would increase the annual capacity of neonatal units. It should be noted that pacifier use does practically no damage in the short term. Therefore, it seems beneficial to introduce pacifiers to preterm newborns already in the hospital, and this should be implemented in clinical practice.

The WHO published the Baby-Friendly Hospital Initiative in 1989, which prohibited the use of pacifiers. In 2018, the Baby-Friendly Hospital Initiative was revised, and the ban on early pacifier use was discontinued because of new research evidence. The new Baby Friendly Hospital Initiative now recommends counseling new mothers about the risks of pacifier use [4]. These risks should not be overestimated. In the future, it should be the caregiver’s own decision whether to introduce a pacifier or not.

We did not have any deviations from the original protocol, which can be regarded as a strength of the study. The limitations of our results are mostly those of the included original studies. The sample sizes were relatively small in all the studies focusing on preterm neonates. Blinding was limited, and most studies described the randomization process poorly. There were some heterogeneities in the interventions as how long the pacifier was advised to be avoided, but as the results of all studies were similar, this should not be an issue in the analysis.

## Conclusion

There seems to be no reason to restrict the use of pacifiers in newborns, as the results of our meta-analysis suggest that they are not associated with breastfeeding duration or success rates. Furthermore, introducing pacifiers to preterm newborns should be considered, as it seems to shorten the time to discharge and the transition from gavage to total oral feeding. Further studies focusing on the factors that improve breastfeeding rates in preterm neonates are needed.

## Supplementary Information

Below is the link to the electronic supplementary material.

Supplementary file1 (DOCX 268 KB)

## Data Availability

Available upon request from the corresponding author.

## Abbreviations

*CI*: Confidence interval
*MD*: Mean difference
*RR*: Risk ratio
