# Supplementary material for: Pacifier use and breastfeeding in term and preterm newborns—a systematic review and meta-analysis
Source: Eur J Pediatr. 2022 Jul 14;181(9):3421–8. doi: 10.1007/s00431-022-04559-9 (PMC9395499; doi:10.1007/s00431-022-04559-9)
Supplement: Supplementary file 1 — Supplementary file1 (DOCX 268 KB) [file 431_2022_4559_MOESM1_ESM.docx]

**Supplementary materials**

**Figure S1**

Figure 1: Flow chart of the review process.

**Identification of studies via databases and registers**

**Identification**

Records identified from*:

Databases (n = 1481)

Records removed *before screening*:

Duplicate records removed (n = 709)

**Screening**

Records screened

(n = 772)

Records excluded**

(n = 728)

Reports sought for retrieval

(n = 44)

Reports not retrieved

(n = 0)

Reports assessed for eligibility

(n = 44)

Reports excluded:

Wrong study design (n = 32)
Double (n = 2)

**Included**

Studies included in review

(n = 10)

**Figure S2**

Risk of bias in individual studies


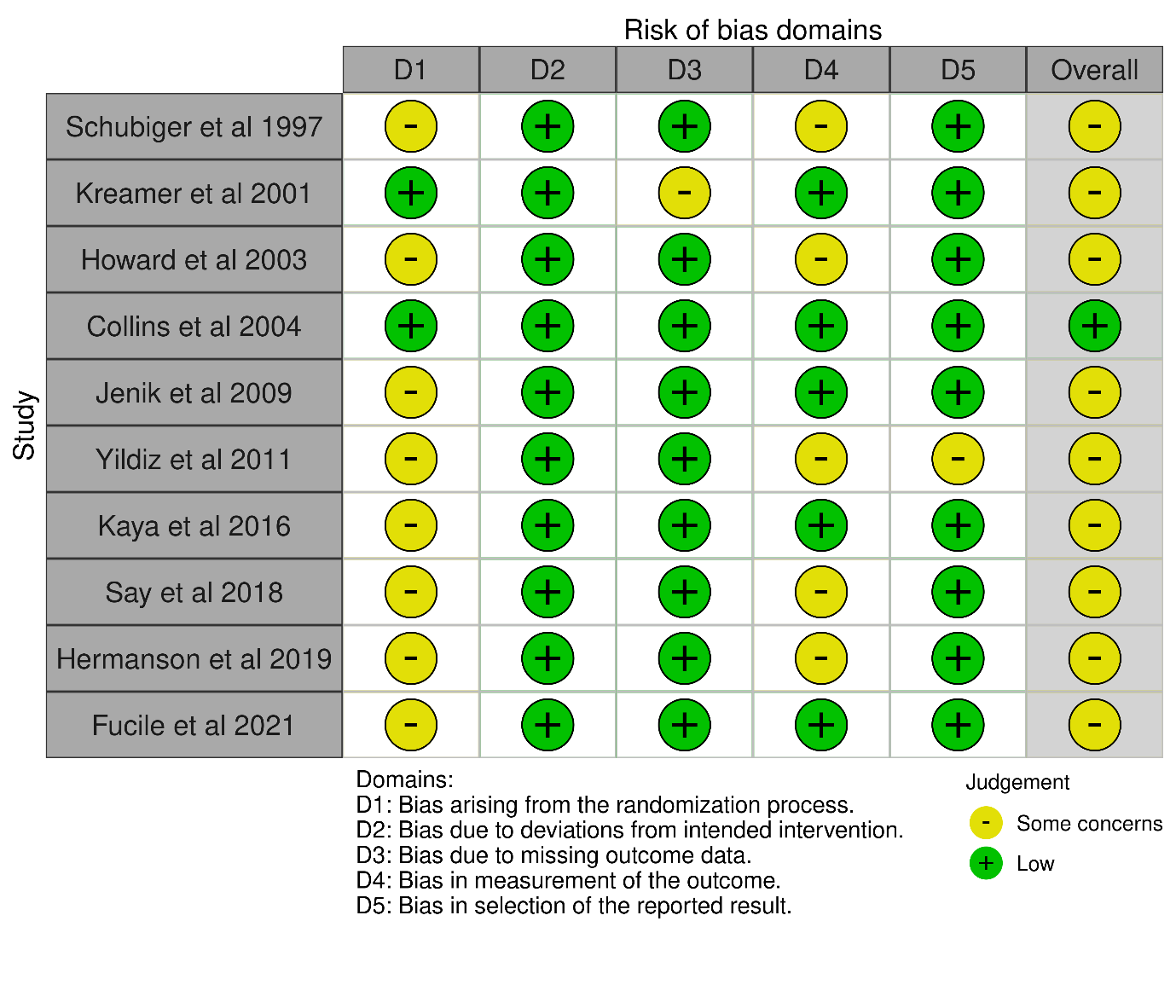


**Figure S3**

Risk of bias in selected domains and the overall risk of bias.


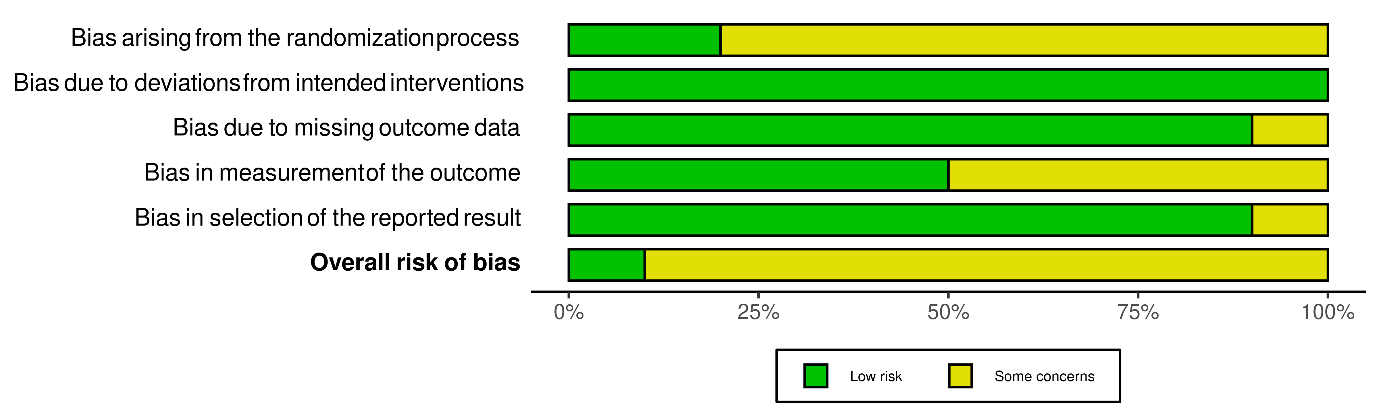


**Table S1** Characteristics of the included studies.

| Study | Country | Study Period | Blinding | Participants total (N) | Intervention | Control | Setting | Inclusion criteria | Exclusion criteria | Main outcome | Secondary outcomes |
| --- | --- | --- | --- | --- | --- | --- | --- | --- | --- | --- | --- |
| TERM NEWBORN | | | | | | | | | | | |
| Schubiger et al 1997 | Switzerland | 1996 | Single | 602 | Pacifiers were forbidden during hospital stay. | Pacifiers without restriction. | Maternity ward | Healthy full-term infants, 37 weeks of gestation, 2750±4200 g | Preterm births, mother does not want to breastfeed | Prevalence of breast-feeding on day 5 | Prevalence of breastfeeding at 2, 4 and 6 months. |
| Kramer et al 2001 | Canada | 1998-1999 | Double | 281 | Recommending avoidance of pacifier for first 3 months. | Pacifier use allowed | Maternity ward | Women who intended to breastfeed for 3 months healthy singleton at least 37 completed weeks 2500 g birth weight | Mother does not want to breastfeed, preterm, under 2500 g | Early weaning (within 3 months) | The frequency and total duration of crying or fussing and the duration of unsoothable crying |
| Howard et al 2003 | USA | 1997-1998 | Single | 700 | Resticted pacifier use for 4 weeks after birth | No restrictions | Maternity ward | Women who intended to breastfeed their infants for at least 4 weeks; had uncomplicated, singleton pregnancies; and were undecided or wanted their infants to use a pacifier; healthy (2200 g or more at delivery, >/=36 weeks’ gestation, with Apgar scores >6 at 1 minute and >7 at 5 minutes) | NICU admission or mother did not want to breastfeed | Time to cessation of overall breastfeeding | The times to cessation of full, and exclusive breastfeeding and breastfeeding-associated maternal and infant problems. |
| Jenik et al 2009 | Argentina | 2005-2006 | Single | 1021 | Recommended not to offer pacifier | Offer pacifier | Neonatal unit | At least 37 completed weeks gestational age 2500 g birth weight exclusively breastfeeding whose mothers reported an intention to breastfeed for at least 3 months | breast problems that could interfere with breastfeeding. Mothers who had decided the use of pacifier | Prevalence of exclusive breastfeeding at 3 months. | Prevalence of exclusive and any breastfeeding at different ages and duration of any breastfeeding. |
| Hermanson et al 2019 | Sweden | 2013-2015 | Single | 239 | Avoid pacifier during the first two weeks. | Recommendation to offer a pacifier from the first day of life | Maternity ward | -women who were primiparous; -intended to breastfeed their infants for at least four months -gave birth to healthy full-term infants (>37 weeks of gestation, birth weight >2500 g). | x | Proportion of breastfeeding at six months. | Proportions of breastfeeding and breastfeeding problems at two and four months |
| PRETERM NEWBORNS | | | | | | | | | | | |
| Collins et al 2004 | Australia | 1996-1999 | Single | 319 | Pacifier during hospital stay | No pacifier in hospital | NICU | Women with singleton or twin preterm infants < 34 weeks’ gestation who wanted to breast feed were eligible for inclusion. | Mother does not want to breastfeed, preterm, unhealthy, under 2500 g, congenital abnormalities precluding enteral feeding. | Proportion of infants fully breastfeed and the proportion receiving any breastfeeding on discard home | Length of hospital stay and prevalence of breastfeeding at three and six months after discharge. |
| Yildiz et al 2011 | Turkey | 2007-2009 | Single | 90 | Pacifier during hospital stay | No pacifier | NICU | Premature infants: • were at 32 (±2) gestation weeks and who did not have sucking reflex  • weighed approximately 1000 gm or more • had Apgar scores >6 • had stable health conditions during the first 24 hours after birth • did not have any congenital malformation that may cause asphyxia and affect respiration and who had spontaneous respiration • did not have cranial bleeding and hyperbilirubinemia that may lead to blood abnormalities • did not have a congenital or acquired malformation related to hearing • had no family members with a hearing loss starting in childhood • were enterally fed via gavage and who could tolerate it • were breastfed • had a mother who was literate in Turkish and who was willing to feed her baby. | No exclusion criteria | Transition period to total oral feeding (hours) | Duration of hospital stay (hours),Weight of discharge (g) |
| Kaya et al 2016 | Turkey | 2013-2014 | Single | 70 | Pacifier use during hospital stay | No pacifier | NICU | Preterm infants 30-34 weeks of gestational age birth weight of ≥1000 g Apgar score of >6 and  not using pacifiers | Infants with a congenital malformation that may cause asphyxia and affect breathing and those with respiratory distress, cranial bleeding, hyperbilirubinemia requiring exchange transfusion, and intestinal anomalies or necrotizing enterocolitis | Time to transition to full breastfeeding | Time to discharge, weight at transition to full breastfeeding, weight of discharge, Sucking skills |
| Say et al 2018 | Turkey | 2016-2017 | Single | 90 | Pacifier use during hospital stay | No pacifier | NICU | Birth weight less than or equal to 1,500 g,  gestational age (GA) younger than 32 weeks,  tolerating at least 100 kcals/kg/day by OG feeding, growth parameters appropriate for GA, and a stable clinical condition. | Preterm infants with congenital anomalies, perinatal asphyxia, prolonged respiratory distress, intraventricular hemorrhage of greater than grade 2 according to the Papille classification,hyperbilirubinemia requiring exchange transfusion, or intestinal anomalies or necrotizing enterocolitis with a Bell stage II to III were excluded. In addition, preterm infants receiving invasive or noninvasive mechanical ventilator support were excluded from the study. | Time for transition to full oral feeding | Time to transition to full breastfeeding, time to discharge |
| Fucile et al 2021 | Canada | 2020 | Single | 33 | Non-nutritive sucking of pacifier once a day for 15 min. | Non-nutritive sucking on an emptied breast for 15 min per day. | NICU | 1) born at less than or equal to 34 weeks gestational age (GA) as determined by obstetric ultrasonogram and clinical examination;  2) appropriate size for their GA; 3) receiving only tube feedings (orogastric or nasogastric); and  4) whose mothers’ intentions were to breastfeed and were pumping milk. | 1) necrotizing enterocolitis (NEC) 2) intraventricular hemorrhage (IVH) grades III and IV; and  3) congenital anomalies (e.g., heart, oral, gastrointestinal etc.) | Exclusive breastfeeding acquisition time to achieve independent oral feeding length of hospitalization | Time to achieve independent oral feeding, length of hospitalization |

NICU = neonatal intensive care unit

**Table S2** Characteristics of the newborns in the included studies.

| STUDY | Number of participants | | Gestational age (mean and standard deviation | | Weight (mean and standard deviation) | |
| --- | --- | --- | --- | --- | --- | --- |
|  | Intervention | Control | Intervention | Control | Intervention | Control |
| Term newborn |  |  |  |  |  |  |
| Schubiger et al 1997 | 294 | 308 | 39.9 (1.4) | 39.9 (1.2) | 3367 (319) | 3404 (348) |
| Kreamer et al 2001 | 140 | 141 | N/A | N/A | 3457 (427) | 3524 (415) |
| Howard et al 2003 | 346 | 354 | 39.8 (0.7) | 39.7 (0.9) | 3612 (456) | 3499 (469) |
| Jenik et al 2009 | 528 | 493 | N/A | N/A | 3690 (477) | 3659 (418) |
| Hermanson et al 2019 | 119 | 120 | N/A | N/A | 3500 (41) | 3520 (44) |
| Preterm newborn |  |  |  |  |  |  |
| Collins et al 2004 | 157 | 162 | 29.5 (2.7) | 29.2 (2.7) | 1344 (488) | 1325 (453) |
| Yildiz et al 2011 | 30 | 30 | 31.9 (1.3) | 31.6 (1.5) | 1469 (266) | 1495 (350) |
| Kaya et al 2016 | 34 | 36 | 32.7 (0.9) | 32.2 (1.5) | 1704 (306) | 1662 (490) |
| Say et al 2018 | 45 | 45 | 29.2 (1.9) | 28.4 (1.8) | 1188 (272) | 1112 (267) |
| Fucile et al 2021 | 17 | 16 | 30.2 (2.4) | 30.4 (2.7) | 1371 (510) | 1491 (478) |
